# Genetic diversity and structure of Asian cowpea germplasm

**DOI:** 10.1038/s41598-025-13511-4

**Published:** 2025-07-31

**Authors:** Ngozi Paulinus Ofem, Nasrein Mohamed Kamal, Sofie Pearson, Tracey Shatte, David Jordan, Emma Mace, Takayoshi Ishii

**Affiliations:** 1 United Graduate School of Agricultural Sciences, Tottori University, 4-101 Koyama Minami, Tottori, 680-8553 Japan; 2 International Platform for Dryland Research and Education (IPDRE), Tottori University, 1390 Hamasaka, Tottori, 680-0001 Japan; 3 Biotechnology and Biosafety Research Center, Agricultural Research Corporation, P.O. Box 30, Khartoum North, Sudan; 4 Queensland Alliance for Agriculture and Food Innovation (QAAFI), The University of Queensland, Hermitage Research Facility, Warwick, Qld Australia; 5 Queensland Department of Primary Industries (DPI), Hermitage Research Facility, Warwick, Qld Australia; 6 Arid Land Research Center (ALRC), Tottori University, 1390 Hamasaka, Tottori, 680-0001 Japan; 7 Chromosome Engineering Research Center, Tottori University, 86 Nishi-Cho, Yonago, Tottori 683-8503 Japan

**Keywords:** Dryland crop, Pulse, DArT-Seq, Population structure, Variation, Dissemination, Genetics, Plant sciences

## Abstract

Cowpea (*Vigna unguiculata* L. Walp., 2n = 2x = 22) is a vital dryland legume crop, renowned for its affordable dietary protein and essential nutrients for humans and animals. Cowpea originated in Africa and spread to various parts of the world through human migration, eventually reaching Asia. However, genetic diversity and structure in Asia cowpea remain poorly understood. This study utilized 6334 SilicoDArT and 14,482 single nucleotide polymorphism (SNP) markers to assess the genetic diversity and population structure of 405 cowpea accessions from 17 different countries, sourced from the National Agriculture and Food Research Organization (NARO) genebank in Japan. We used population structure, principal component analysis, discriminant analysis of principal components, and phylogenetic tree analysis to group the accessions into two main genetic populations. The accessions were further classified into six subgroups of African and Asian populations, corresponding to the geographical origins of the accessions. South Asian accessions showed the highest differentiation, with Nepalese accessions forming a distinct group along with Japanese accessions, highlighting that the rich genetic resources preserved within these regions may harbor valuable traits for breeding. In contrast, Southeast Asian and West African accessions exhibited low to moderate differentiation, suggesting recently shared genetic ancestry. AMOVA demonstrated that most genetic variation existed within accessions, while variation between populations was minimal. These findings highlight the rich genetic potential within the Asian cowpea germplasm, particularly in Nepalese and Japanese accessions. This study provides critical insights into breeding strategies aimed at enhancing the adaptability and productivity of cowpea in diverse environments.

## Introduction

Cowpea (*Vigna unguiculata* (L.) Walp., 2n = 2x = 22), a member of the Fabaceae family, is a vital annual crop cultivated for grain, vegetable, and fodder purposes in tropical and subtropical regions, particularly in Africa, Latin America, Asia, Southern Europe, and the United States^1^. Cowpea plays a critical role in food security and nutrition, especially in sub-Saharan Africa and other developing regions^2–5^. Cowpea is rich in carbohydrates (64%), protein (25%), and essential micronutrients such as iron, zinc, calcium, and magnesium, making it an important dietary component^3,6,7^. Besides human consumption, cowpea serves as a highly nutritious feed for livestock^8,9^. Recent efforts in breeding have focused on developing dual-purpose varieties with high grain and fodder yields^10^.

Cowpea is well adapted to heat and drought-prone environments and thrives in low-fertility soil due to its nitrogen-fixing ability through symbiosis with *Rhizobia*^11,12^. It is commonly intercropped with cereals and used in crop rotation systems to improve soil fertility and agricultural sustainability^13,14^. West Africa remains the largest producer of cowpea, contributing significantly to global production at 7.8 out of 8.9 million tons of dry cowpea cereal in 2021, with Nigeria leading in output, followed by other nations in sub-Saharan Africa^15^.

The genetic diversity of cowpea is crucial for breeding improved varieties. However, cultivated cowpea has a narrow genetic base due to its self-pollinating nature and domestication bottlenecks^4,10,16,17^. The center of diversity for cultivated cowpea is in West and East Africa. The oldest evidence that cowpeas existed in West Africa is obtained from the carbon dating specimens from the Kintampo rock shelter in Central Ghana^18^. Wild cowpea is believed to have originated in Southern Africa, with *Vigna unguiculata* ssp. *dekindtiana* (Harms) Verd., recognized as its ancestor. This subspecies is extensively found throughout Africa^19^. Previously classified into five groups based on traits like flower color, pod length, and seed color^20–22^, cowpea has evolved into three main subspecies. *Vigna unguiculata* ssp. *unguiculata* is the most common, grown for seeds in sub-Saharan Africa, featuring a bushy growth habit, determinate growth, and short pods (10–22 cm) with white, brown, or black seeds^23^. *Vigna unguiculata* ssp. *sesquipedalis,* the asparagus bean or “yard-long bean,” is cultivated in China, South Asia, and Southeast Asia for its long edible pods (30–50 cm). It has an indeterminate growth habit, climbing stems, and seeds that vary in color, including red, black, brown, or purple^19,24,25^. *Vigna unguiculata* ssp. *cylindrica* is characterized by small, round, dark or gray seeds and is primarily used as a fodder crop^26^.

Genetic diversity within crop populations provides insights into their evolutionary trajectory and breeding potential. Studies have explored cowpea’s domestication and global dissemination through genetic analysis^27–30^. Historically, cowpea spread from West and East Africa to South and Southeast Asia and Europe around 200–300 BC^13,31^. The genetic structure of cowpea populations is shaped by evolutionary forces such as mutation, gene flow, genetic drift, and natural selection^32,33^. The highest genetic diversity is found in primary centers of diversity, but secondary centers of diversity can emerge in regions where the crop has been extensively cultivated^34,35^. Herniter et al.^13^ proposed that cowpea entered Asia through the “Sabaean lane” in Yemen, reaching India by 1500 BCE before spreading to Southeast Asia. After its introduction into Asia, cowpea underwent further diversification in India and Southeast Asia, which resulted in the development of ssp. *sesquipedalis*, known for its elongated pods used as a vegetable, and the ssp. *biflora* (*cylindrica*) as a valued fodder crop^20,21,36,37^.

Despite the extensive domestication history of cowpea in Asia, genetic studies on Asian germplasm remain limited. Many genetic diversity studies have focused on Africa due to its role as the primary gene pool, as well as the presence of wild progenitors and diverse landraces^3,38–40^. IITA genebank houses around 17,000 accessions of cowpea which have been extensively used for the development of over 800 improved cowpea cultivars^41^. Cowpea cultivation in Asia dates back over 2000 years with India and China playing significant roles in its history. In China, records indicate at least 500 years of cowpea cultivation^31,42–44^. In Japan, cowpea populations thrive in ruderal habitats across the country, particularly in the southern regions, where temperatures range from 28 to 30 °C during rainy seasons which create optimal conditions for their growth^45^. Cowpea seeds in Japan typically come in black or red varieties and are traditionally used in culinary dishes like rice cakes, zenzai (sweet red bean soup with pieces of rice cakes), and sekihan (steamed rice mixed with red beans)^46^. This culinary tradition mirrors that of the azuki bean (*Vigna angularis*), which belongs to the same genus and is cultivated in Japan. The preference for seed coat colors varies across regions, with African regions favoring brown and white seeds^47–50^.

This study analyzed 410 cultivated cowpea accessions and wild relatives from 18 countries, sourced from the National Agriculture and Food Research Organization (NARO) Genebank Japan. We utilized DArT-Seq markers to examine the genetic diversity and population structure of Asian cowpea germplasm. Our results provide valuable insights into the genetic resources available in Asia and the dissemination of cowpeas following their introduction to the region.

## Materials and methods

### Plant materials

A total of 410 accessions of cowpea (*Vigna unguiculata* L. Walp.) were used in this study. Of these, 405 accessions were obtained from the NARO Genebank in Japan, and the remaining 5 from our collection. These accessions were distributed across 18 countries, mostly in Asia. Of the cowpea accessions, 386 belong to subspecies *unguiculata* (including one accession from *V. unguiculata* ssp. *stenophylla*, a wild relative of cowpea native to Botswana), 21 belong to ssp. *sesquipedalis* and 3 belong to ssp. *biflora* (*cylindrica*). The 349 accessions representing Asia originate from Japan (126), Nepal (103), Pakistan (45), Cambodia (18), Laos (15), Sri Lanka (13), Myanmar (10), China (8), Thailand (7), and Malaysia (4). The 59 African cowpea accessions originate from Nigeria (23), Ghana (16), the Republic of Benin (10), Sudan (5), Ethiopia (4), and Botswana (1). Europe and Oceania were represented by one accession each from Russia (1) and Papua New Guinea (1). In addition, five accessions in our collection incorporate the Nigerian accessions IT86D-1010 and IT97K-499-35 as cowpea reference genomes in phylogenetic analyses (Table S1 and Fig. S1).

### DNA extraction and sequencing

All cowpea seeds were germinated in the field of the Arid Land Research Center, Japan, and a single leaf was collected from each plant. The collected leaves were punched directly into the 96-sample tube, then stored in an ultra-low humidity desiccator model TDC-162-HYP (Tolihan Corporation, Tokyo, Japan) for one week at room temperature. Following the drying period, the leaves were ground with two 4 mm zirconia beads with a plate machine model Schwingmühle Tissue Lyser 2 (QIAGEN GmbH, Hilden, Germany) for 1 min. Subsequently, the processed 96-well plates were shipped to the Diversity Array Technology (DArT) company in Australia. At DArT, DNA extraction was performed, and Next-Generation Sequencing (NGS) data was generated for further analysis.

### SilicoDArT and SNP calling and data filtering

Quality control measures were applied to the raw data, including filtering for high-quality markers and trimming using DArTSeq™ technology. The genotyping company, DArT, generated 6334 polymorphic SilicoDArT markers and 14,482 SNP markers across all 410 accessions from 18 countries (Table S1). Markers (SilicoDArT and SNPs) with missing data and rare SNPs/markers with < 5% minor allele frequency (MAF) were eliminated to ensure data reliability. SilicoDArT markers exhibited ≥ 95% reproducibility and a call rate value of ≥ 81%, with an average of 95%, including 1752 markers with no missing data. SNP markers had an average of 99% reproducibility with a mean call rate of 80%, ranging from 20 to 100%, with approximately 9903 markers exceeding 90%. After filtering the missing data, 4860 SNP loci were retained. Due to the high percentage of monomorphic markers, the 1752 SilicoDArT and 4860 SNPs were retained, accounting for 26.5% and 33.6% of the total markers, respectively. These markers were chosen based on a stringent polymorphic information content (PIC) threshold of 0.19. The final curated dataset of 6612 markers was used for genetic diversity and population structure analyses.

### Population structure analysis

For the genetic diversity analysis, we utilized a combined 6612 markers in a subset of 405 cultivated cowpea accessions, all of which had complete genotype data and marker information. Our collection of five accessions was excluded from this analysis because two of them lacked passport information in the database, and to accurately assess genetic diversity distribution based on the newly sequenced subset data. Population structure was inferred to assess the distribution of genetic diversity among accessions using the model-based program STRUCTURE v2.3.1 https://web.stanford.edu/group/pritchardlab/software/structure_v.2.3.1.html^51^. To determine the optimal number of populations (*K*) and to capture the overall structure within the dataset, we conducted ten independent runs for each simulated value of K, ranging from 1 to 10. Each run consisted of a burn-in period of 50,000 iterations, followed by a run length of 100,000 Markov Chain Monte Carlo (MCMC) iterations. Subsequently, the putative optimal *K* was determined using the pophelper v2.3.1 R package^52^. using the delta *K* (∆*K*) method^53^. Individuals were assigned to clusters if their proportional membership probability (Q) was at least 80%. This was determined using Q-values. To visually represent the population structure, bar plots were sorted by Q-values according to the optimal *K* identified in the analysis.

### Principal component analysis (PCA) and phylogenetic tree analysis

The genetic variation among geographical locations and regions within the subset of 405 cowpea accessions was analyzed using the two-dimensional PCA plot generated with the R built-in function “prcomp”. First, an identity-by-state (IBS) distance matrix was obtained using TASSEL 5.0 https://www.maizegenetics.net/tassel^54^ and then converted for use in R (version 4.3.3) https://www.r-project.org/. The PCA analysis focused on these 405 cowpea accessions, utilizing 6612 markers. Additionally, the genetic distance among all 410 accessions was calculated in MEGA 11 https://www.megasoftware.net/^55^. To comprehensively infer genetic relationships within the accessions in the phylogenetic analysis, our collection of five accessions, consisting of four cultivars and one wild relative (*V. unguiculata* ssp. *stenophylla*), was incorporated into the 405-accession subset. In the phylogenetic tree construction, the wild relative served as the out-group, and the tree was built using the maximum likelihood method. The bootstrap method with 1000 replications and the Tamura-Nei model was employed for phylogeny testing, with rates among sites provided for an automatic initial tree (neighbor-joining). Furthermore, the tree color, shape, and branch representation of each genotype were determined based on the cluster (Q) derived from STRUCTURE analysis using iTOL software (v5) https://itol.embl.de/^56^.

### Discriminant analysis of principal components (DAPC)

Since cowpea is a self-fertilizing and highly inbred taxon, we also performed a discriminant analysis of principal components (DAPC) on the subset of 405 cowpea accessions to better distinguish groups contributing to the overall genetic variation identified by the PCA. To determine the most suitable clusters within the cowpea germplasm, we applied DAPC, a multivariate technique that uses sequential means of *K* (number of clusters) and model selection to infer and characterize clusters in genetically related populations^57^. The optimal *K* value was determined as the minimum number of clusters at which the Bayesian information criterion (BIC) exhibited minimal change (increase or decrease), allowing for a more refined analysis of population structure^58^. K-means clustering and DAPC were performed using the *adegenet* package v2.1.10^57^ within the R programming environment v4.3.3^59^.

### Genetic diversity analysis

For the genetic diversity analysis, we utilized PowerMarker software (v3.25) https://brcwebportal.cos.ncsu.edu/powermarker/^60^ to analyze 6612 markers. Key diversity metrics, including PIC (polymorphism information content) and MAF (minor allele frequency), were calculated, including their minimum, median, mean, and maximum values. Additionally, an analysis of molecular variance (AMOVA) was performed to assess population differentiation within accessions and between populations using GenAlEx 6.5 https://biology-assets.anu.edu.au/GenAlEx/Welcome.html^61^ with 999 permutations for statistical significance testing. To ensure accurate diversity representation, we excluded one accession each from Europe and Oceania due to their small sample sizes, which were insufficient for meaningful diversity assessment. A minimum of four accessions per region was required to perform AMOVA using the GenAlex package.

## Results

### Analysis of population structure

**Fig. 1:**
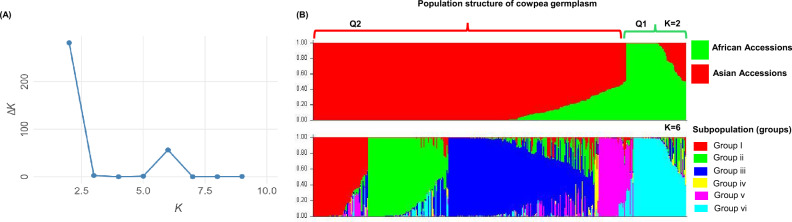
Structure analysis was analyzed with 6612 markers. (**A**) The optimal number of *K was* determined using Δ*K* calculated from 2 to 9. (**B**) Population structure classification of the subset of 405 cowpea accessions using membership probability (Q-values) at *K* = 2 (Q1: green, Q2: red) and *K* = 6 (groups; I: red, II: green, III: blue, IV: yellow, V: pink, and VI: blue-green), and the distribution of the accessions to different populations or subpopulations is indicated by the colour code.

The population structure of the subset of 405 cowpea accessions was inferred based on 6612 markers. Based on the ranked Q-values from the STRUCTURE analysis, an accession was assigned to a genetic group if membership assignment to a group was higher than 79%, while a lower score indicated admixture. The ∆*K* method used to determine the optimal number of genetic clusters revealed two peaks (Fig. 1A), with *K* = 2 best fitting the data. At *K* = 2, STRUCTURE analysis identified two distinct genetic populations within the cowpea germplasm, designated as groups Q1 and Q2 (Fig. 1B). The distribution of the cowpea accessions among the two populations revealed that group Q2 (red) had the highest percentage of membership (68.9%) with 279 accessions, while group Q1 (green) had the lowest percentage of membership (10.9%) with 44 accessions. However, the inferred ancestry indicated that 82 accessions (20.2%) were admixed (Fig. 1B), representing the sum of variation from the two groups.

Group 1 (Q1, green) consisted of accessions sourced from diverse regions, namely West, East, and Northeast Africa, as well as East, South, and Southeast Asia. Q1 included accessions from Cambodia, Ethiopia, Ghana, Nepal, Nigeria, Japan, Pakistan, Sri Lanka, Sudan, and Thailand. Notably, 36% of the total accessions originating from Africa, particularly West Africa, were categorized within Q1 (Table 1). Conversely, Group 2 (Q2) encompassed most of the accessions from East, South, and Southeast Asia, along with a few from Europe, East, and West Africa. Accessions in Q2 were derived from Cambodia, China, Ethiopia, Japan, Laos, Malaysia, Myanmar, Nigeria, Nepal, Pakistan, Sri Lanka, Sudan, Russia, and Thailand (Table 1). This distribution underscores the significance of these groups in representing their respective regions. Additionally, Q1 excluded accessions from China, Laos, Malaysia, and Myanmar, as these countries comprised accessions of ssp. *sesquipedalis*, which were solely found in Q2. One accession of ssp. *sesquipedalis* sourced from Oceania was identified in a genetic admixture with other accessions in Q2. In the ssp. *cylindrica* collection, two accessions from Pakistan were clustered in Q1, while one accession from Nepal showed a mixture of both populations. Considering the share of ancestral populations by each country (Fig. 1B), the Nepalese, Japanese, and Pakistani germplasms were the only ones that belonged predominantly to ancestral population 2, with 87.4%, 84.6%, and 82.2% accessions in Q2. Overall, admixed ancestry was highest in Q2 (14.8%) and lowest in Q1 (5.4%), indicating a higher degree of genetic mixing in Q2 compared to Q1, and reinforcing the evidence supporting the introduction of cowpea from Africa to Asia.

**Table 1:** Population structure analysis of cowpea accessions in each population by number and percentage throughout the eight (8) regions.

Regions	Number of accessions in each population			Percentage of accessions in each population			Total number of accessions in each region
	Q1	Q2	Admixture	Q1	Q2	Admixture	
East Asia	4	110	18	3.0	83.3	13.6	132
South Asia	14	128	19	8.7	79.5	11.8	161
Southeast Asia	5	36	13	9.25	66.6	24.1	54
East Africa	1	2	1	25.0	50.0	25.0	4
Northeast Africa	4	1	0	80.0	20.0	0	5
West Africa	16	1	30	34.0	2.1	63.8	47
Europe	0	1	0	0	100	0	1
Oceania	0	0	1	0	0	100	1

Population substructure analysis across different regions estimated with peak *K* = 6 (Fig. 1A). The subpopulation structure analysis split the accessions into six distinct groups. Group 1 (red) comprised 60 accessions from Benin, Cambodia, Ethiopia, Ghana, Japan, Laos, Nepal, Nigeria, Pakistan, Thailand, Sri Lanka, and Sudan; group 2 (green) consisted 87 accessions from China, Nepal, Japan, Malaysia, and Myanmar; group 3 (blue) included 159 accessions from Cambodia, China, Japan, Laos, Nepal, Nigeria, Malaysia, Myanmar, Sri Lanka, Pakistan, Papua New Guinea, and Thailand; group 4 (yellow) contained 4 accessions from China, Nepal, Nigeria; group 5 (pink) encompassed 38 accessions from Benin, Cambodia, Ethiopia, Ghana, Japan, Nigeria, Pakistan, and Russia; and group 6 (blue-green) comprised 57 accessions from China, Ethiopia, Japan, Nepal, Pakistan, and Sudan. The grouping indicated that groups 1 and 5 predominantly represented the African population, while groups 2, 3, 4, and 6 were predominantly Asian. Notably, most accessions from Nepal and Japan were distinctively clustered in groups 2 and 3, suggesting a significant genetic divergence from other regions.

### Principal component analysis (PCA)

**Fig. 2:**
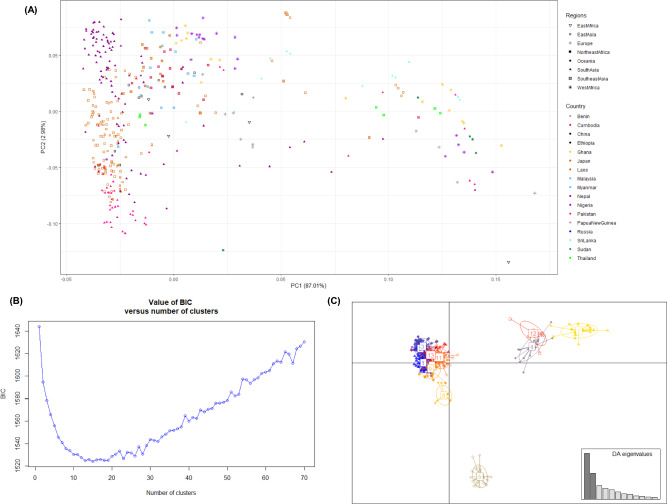
Principal Component Analysis (PCA), Discriminant analysis of principal components (DAPC) scatter plot based on 6,612 markers. (**A**) PCA analysis with the subset of 405 accessions along the PC1 and PC2 axes. Shapes and colors represent individual accessions grouped by region and country, respectively. (**B**) Bayesian Information Criteria (BIC) for different levels of *K* showing the presence of at least six different genetic clusters. (**C**) DAPC shows the grouping of cowpea germplasm into six main genetic clusters.

The two-dimensional PCA plot captures 89.9% of the overall variation, with the first principal component (PC1) accounting for 87.0% and the second principal component (PC2) accounting for 2.98% of the variation (Fig. 2A). Overall, the accessions were continuously distributed along these principal components, suggesting frequent admixture among population groups. In the PC2 plot, Asian accessions were positioned along the vertical axis, while the African accessions were along the lower (horizontal) axis in PC1. This separation suggests that the Asian germplasm originated from a subset of African genetic diversity, making it relatively narrow compared to African. However, within the Asian population, there is a significant genetic variation, particularly among accessions from Nepal, Japan, and Pakistan, which formed distinct groups within Q2. The overlap between Q1 and Q2 indicates significant introgression between these clusters. Additionally, these three cultivars were clustered within cowpea accessions collected from landraces, further supporting genetic mixing.

**Fig. 3:**
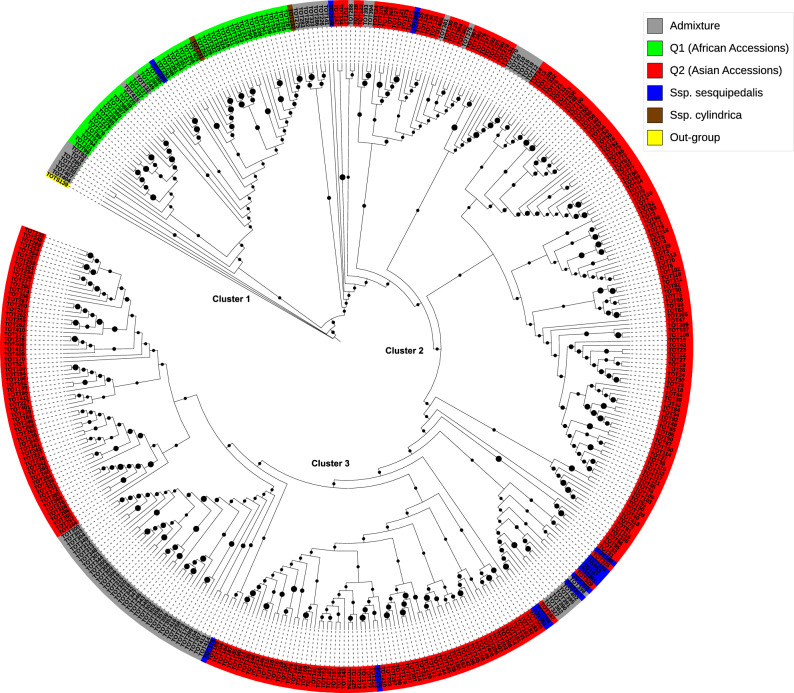
Maximum-likelihood phylogenetic tree illustrating the evolutionary relationships among all 410 cowpea accessions analyzed with 6612 markers. The mapped markers assign individuals to two populations (Q1 and Q2), which correspond to their geographical and cultivar-specific origin or domestication history. Green and red colors correspond to Q1 and Q2, respectively, as defined by the phylogenetic tree and population genetic structure. Black dots indicate bootstrap values ranging from 0 to 1.

The existence of different genetic groups and the assignment of accessions into groups using the DAPC and Bayesian Information Criterion (BIC) approach agreed with the model-based population structure analysis. The DAPC scatter plot demonstrated a clear division of accessions into 13 subclusters distributed across six distinct groups ranging from 1 to 6 (Fig. 2B). Increasing the parameter *K* in this genetic analysis significantly improved the identification and distinction of distinct groups within the Asian and African gene pools, reflecting the impact of strong geographic trends on genetic variations. Similarly, the BIC curve generated from *K*-means clustering rapidly declined from *K* = 1 to *K* = 6, suggesting the existence of at least six genetic groups and a maximum of 13 subclusters (Fig. 2B). At *K* = 6, the scatter plot (Fig. 2C) portrays the divergence among all identified genetic clusters. Notably, group 1 encompassed 8 subclusters (1, 2, 3, 6, 9, 10, 11, and 13), which demonstrated a high degree of overlap, while group 2 contained one cluster (subcluster 8). Both group 1 and group 2 were specific to the Asian gene pool (Q2). Conversely, groups 3, 4, and 6 contained one subcluster each (4, 12, and 7), characterized by the African gene pool (Q1). Group 5 consisted of all admixed individuals (Fig. 2B). Despite the clear structuring, no group exclusively consisted of accessions from the same country or region. However, there were instances where all accessions from the same country belonged to the same group, as seen with the Nepal accessions. This pattern was consistent with the groupings observed on the phylogenetic tree (Fig. 3). The analysis also confirmed the segregation of the subspecies *sesquipedalis* subcluster within the Asian gene pool.

### Genetic differences and distance among populations

For 403 of the 405 cowpea accessions, 6612 markers were combined to perform a region-based AMOVA to assess the genetic variation between and within populations. The results of the AMOVA revealed that 7.0% of the total genetic variation was attributed to differences among the two main populations, while the remaining 93.0% was found within populations (Table 2). A second AMOVA, conducted using the 15 evaluated populations based on their country of origin, showed that 20.0% of the overall variation was due to differences among all populations and 80.0% resided within populations (Table S2). This indicates that most genetic diversity resides within individual populations. The average polymorphism information content (PIC) was 0.19. The genetic dissimilarity between the accessions estimated from the combined markers ranged from 0.0004 to 0.15, with an overall average distance of 0.02.

**Table 2:** Analysis of molecular variance (AMOVA) results partitioning genetic variation among regions and within cowpea accessions.

Source of variation	df	SS	MS	Est. Var	%	*p*-Value
Among Pops	1	485.745	485.745	4.402	7%	0.001
Within Pops	401	24,559.260	61.245	61.245	93%	0.001
Total	402	25,045.005		65.647	100%	
Nm	6.96					

*df* degree of freedom, *SS* sum of squares, *MS* mean square, *Est. Var.* estimated variance, % percentage variance, *Nm* gene flow.

### Cluster analysis

The maximum likelihood phylogenetic tree revealed three major clusters (1–3) among the full cohort of 410 cowpea accessions, broadly separating them into two populations: Q1 (green) corresponding to Cluster 1 and mainly composed of African accessions, and Q2 (red) encompassing Clusters 2 and 3 and primarily consisting of Asian accessions. A distinct group of admixed individuals (grey) and a wild outgroup (yellow) were also identified. Within Q1, African accessions formed several subclusters, suggesting narrow genetic divergence within the group. Q2 (red) showed greater substructure with multiple smaller clusters, particularly among *V. unguiculata* ssp. *sesquipedalis* (blue), which clustered closely with accessions from Japan, China, South, and Southeast Asia, indicating a strong regional association. This pattern suggests a shared ancestry between *ssp. sesquipedalis* and cowpea accessions from East and Southeast Asia, where the crop was reported to have diverse landrace forms^62^. Distinct subclustering within ssp. *sesquipedalis* suggests its genetic divergence from cultivated cowpea types, which supports the evidence of separate domestication in Asia, due to intense selection for pod quality traits favourable to use as vegetables and characteristic climbing growth habit, following introduction from Africa^63^. Furthermore, the close clustering of Japanese, Chinese, and Southeast Asian accessions suggests possible historical dissemination of cowpea germplasm from these regions into Japan (Fig. S2). A single breeding line of *sesquipedalis* grouped with Thai ssp. *unguiculata* within Q1, indicating a potential breeding-related or historical introduction from Asia. A separate cluster of Nepalese accessions (top left of Cluster 3) reflected regional differentiation. Ssp. *cylindrica* accessions (brown) were exclusively grouped within Q1, subclustering with ssp. *unguiculata*. The wild out-group, ssp. *stenophylla*, was positioned at the root of the tree, highlighting its genetic divergence from cultivated cowpea species (Fig. 3).

## Discussion

Investigating genetic relationships and diversity within plant varieties is essential to promote the sustainable use of genetic resources^29^. Although other studies have provided global or Africa-centric insights into cowpea diversity^4,9,38–40,43,64,65^, this study fills a major gap by focusing on Asian genetic resources collected from 18 countries across multiple continents. Only a few studies have investigated the detailed genetic diversity of Asian cowpea genetic resources.

Analyzing population structure is crucial in identifying the genetic foundation of complex traits for association studies^66^. In this study, we examined 410 cowpea accessions selected for their agronomic trait diversity, specifically seed coat color and pod length, representing genetic variation across 18 countries. Our analysis of population structure revealed that NARO cowpea germplasm is genetically divided into two geographically distinct subpopulations. The first subpopulation (Q1) includes accessions primarily from West Africa, Northeast Africa, and South Asia, while the second subpopulation (Q2) comprises accessions from East Africa, South Asia, Southeast, East Asia, and Europe. At *K* = 2, the analysis identified two main gene pools as indicated by the peak of delta *K* (∆K), highlighting clear genetic differentiation within the cowpea population. Further analysis at *K* = 6 revealed additional sub-structuring, dividing the accessions into six distinct groups, likely representing sub-division events among the three cultivated subspecies: grain, fodder, and vegetable cowpeas.

The Q1 subpopulation contained two subspecies: *Vigna unguiculata* ssp. *unguiculata*, which is cultivated for seeds in sub-Saharan Africa and exhibits white, brown, and black seed coats with pod lengths of 10–22 cm, and *Vigna unguiculata* ssp. *cylindrica*, which features black and red seed coats. Q2 consisted of accessions from ssp. *unguiculata* and ssp. *sesquipedalis*, with the latter distinguished by its red, black, brown, and purple seeds and pod lengths (30–50 cm).

The levels of admixture varied between the two subpopulations, with Q1 showing 5.4% admixture and Q2 displaying 14.8%, suggesting potential gene flow or shared ancestral origins. The mixed geographical distribution of these genetic groups was further supported by findings from the DAPC and BIC analyses. The higher level of admixture in Q2 is likely influenced by historical and breeding-related factors. As cowpea spread from Africa to Asia, it diversified through natural and human-mediated selection, leading to the development of ssp. *sesquipedalis*, an early maturing cultivar valued for its long and tender pods used as vegetables. Asia’s long history of cowpea cultivation, combined with traditional farming practices, seed exchange networks, and farmer-driven selection, facilitated genetic mixing among landraces and cultivars. Additionally, modern breeding programs and germplasm exchange between regions have further contributed to admixture by incorporating diverse parental lines for cowpea improvement.

The geographical distribution of the identified populations was consistent with the results obtained from the PCA and phylogenetic tree based on genetic distance analysis. The delineation of accessions in Q1 primarily consisted of those from Western and Northeast Africa, along with East, South, and Southeast Asia. In contrast, Q2 included accessions from East, South, and Southeast Asia, East Africa, and Europe, and these results remained consistent across multiple analyses. Notably, West African accessions were predominantly grouped in Q1, with minimal representation in Q2, except for a single Nigerian accession. This distinct clustering suggests a unique genetic signature or ancestry for West African accessions compared to other regions.

While no population was entirely composed of accessions from the same region, certain regions showed a strong tendency to fall into either group Q1 or Q2. West African accessions (Benin and Ghana), Southeast Asia (Laos, Malaysia, and Myanmar), and Chinese accessions, predominantly clustered within a single group, indicating admixture ancestry. The clustering of accessions into Q1 or Q2 suggests a complex interplay of genetic diversity, history of genetic exchange, migration, and admixture among the studied populations. Most cowpea accessions exhibited mixed parental lines, particularly those from West Africa, as well as a single accession from Papua New Guinea, except for those from Sudan, Russia, and Thailand. The Papua New Guinea accession classified as ssp. *sesquipedalis* was identified as a breeding line and remained unassigned to a specific cluster due to its mixed genetic background. This suggests a potential historical or breeding-related exchange from Asia. The Russian accession belonged to Q2, while Sudanese and Thai accessions were found in Q1 and Q2. However, the limited number of accessions contributed from Russia and Sudan (one to five) may not adequately capture the full diversity of cowpea in these areas.

Compared to previous studies analyzing global cowpea germplasm collections, which identified genetic divergence between West and East African germplasm^9,61,62,64^, our findings reinforce this distinction as the primary structuring factor in cowpea diversity. Despite the limited number of East African representatives, Ethiopian accessions grouped in Q2 alongside Asian accessions, suggesting a genetic similarity between these regions. The confinement of West and Northeast African accessions to Q1, along with evidence of a mixture between West African accessions and Asian accessions, further underscores recently shared ancestry lines between these populations. Among the 347 Asian accessions, 79.3% and 6.6% were assigned to Q2 and Q1, respectively, indicating long-term coexistence of both genetic backgrounds in Asia. Importantly, the clear distinction between cowpea accessions from Asia and Europe and those from West Africa remains a significant and consistent finding.

Further analysis using DAPC and BIC identified six main groups (1 to 6), which were further divided into 13 subclusters, reflecting geographic influences. At *K* = 6, the scatter plot distinctly illustrates divergence among these identified genetic clusters. Group 1, the most diverse, contained 8 subclusters, indicating significant genetic variation likely due to its broad geographic distribution and extensive genetic exchange. Group 2, in contrast, comprised a single, more homogenous cluster predominantly associated with the Asian gene pool (Q2). Groups 3, 4, and 6, each containing one subcluster, showed limited genetic variation and were primarily associated with the African gene pool (Q1). Group 5 displayed admixed genetic profiles, suggesting gene flow between populations. Within the Asian gene pool, DAPC analysis confirmed a distinct subcluster of ssp. *sesquipedalis* and other unique subclusters for Nepalese and Japanese accessions. The distinct subcluster of Nepalese is likely attributed to the diverse and extreme environmental conditions in the Hindu Kush Himalaya Range, which promotes adaptation and genetic differentiation in crops like cowpeas^66–68^. The genetic divergence found in Nepal and Japan may result from distinct climates. Nepal’s high-altitude, subtropical, and temperate conditions, and Japan’s cooler, high-precipitation environment, may have shaped unique stress-adaptive traits in cowpea from these regions. Additionally, Nepal and Japan both preserve landraces, however, Japan also emphasizes targeted breeding, further reinforcing genetic distinctiveness. The high-altitude and high-precipitation environments are distinct from the area of domestication in Africa, suggesting that Nepalese and Japanese genetic groups may harbor environmentally adaptive alleles.

Despite the identified subclusters, the genetic variation within the African population was low to moderate, suggesting that geographical proximity and exchange of genetic materials within West African regions have reduced genetic isolation. Although no single group exclusively comprised accessions from the same country or region, South Asian accessions showed a tendency to cluster together, reinforcing regional genetic coherence, consistent with phylogenetic tree groupings. The low to moderate genetic variation observed, particularly within the African population, where genetically similar individuals are clustered into distinct gene pools, may limit the availability of novel alleles for cowpea breeding programs. Consequently, crossing members of different populations, such as African and Asian accessions, is expected to enhance genetic diversity and produce more valuable progenies. As Huynh et al.^61^ previously noted, breeding programs often operate within narrow genetic pools, and without the active introduction of new germplasm, genetic variation declines over time, constraining both immediate and long-term genetic progress.

Based on the maximum-likelihood method, the phylogenetic analysis among the 410 cowpea accessions revealed a close genetic relationship between ssp. *cylindrica* and ssp. *unguiculata,* whereas ssp. *sesquipedalis* formed distinct subclusters. This finding is consistent with previous research findings by Wu et al.^69^. Despite their genetic distinction, both ssp. *sesquipedalis* and ssp. *cylindrica* have been traditionally cultivated in Asia for different agricultural purposes^26,70^. Ssp. *cylindrica* is primarily used as livestock feed or a cover crop to control weed growth^71^. The hierarchical clustering categorized the accessions into two main sections: Q1 (green) and Q2 (red), with additional admixed individuals (grey) and an outer group (yellow). Ssp. *sesquipedalis* accessions were exclusively found in Q2, forming distinct, blue-marked clusters. This exclusivity indicated a clear genetic separation from other subspecies, confirming their genetic separation from other subspecies. In contrast, ssp. *cylindrica* was found exclusively in Q1, reinforcing its genetic distinctiveness. Ssp. *unguiculata* exhibited greater genetic diversity, with accessions distributed across both Q1 and Q2.

Geographical clustering was also observed with accessions from the same region often grouped closely. For instance, accessions from Nepal, Pakistan, and Sri Lanka (South Asia) formed close clusters (Cluster 3), as did accessions from Southeast Asia. This suggests that regional breeding practices and environmental factors have influenced genetic variation. Within Q1 and Q2 populations, several small clusters were identified, often characterized by shared ancestry. Additionally, some accessions of ssp. *unguiculata* and ssp. *sesquipedalis* exhibited mixed parental ancestry, highlighting the complex genetic dynamics and historical gene flow within the cowpea population.

We proposed the historical migration patterns of cowpeas into Asia based on genetic analysis. The investigation focused on tracing the migration routes of Asian cowpeas introduced from Africa, employing phylogenetic relationships and gene flow analysis conducted with MEGA 11. The results suggested that the flow of genetic material in cowpea germplasm towards Asia and other regions can be traced back to its origins in West and East Africa, corroborating earlier findings by Xiong et al. (2016). Cowpea domestication in Africa predates 1500 BC^72,73^, with recent findings suggesting a dual center of domestication^37^. It is suggested that the cowpea was first introduced into Asia around 1500 BC during the Neolithic period, establishing a secondary center of origin in India. As cowpea spread through Asia due to different climatic conditions, the selection for fresh pod consumption led to the emergence of the cultivated group *V. unguiculata* ssp. *unguiculata* cv. *sesquipedalis* in regions like Thailand, China, the Philippines, and India^5,37,59,61,74,75^. Findings from our study showed that the few accessions contributed from Ethiopia clustered with South Asian accessions in Q2, indicating a similar ancestry and the possible early introduction of East African germplasm into this region. Most likely, cowpea went to South Asia at some point from East Africa, possibly through trade links^70^. The distribution of cowpea in Asia was mainly from India, South, East, and Southeast Asia, with Japanese accessions primarily from East and Southeast Asia, consistent with previous findings^76,77^. The phylogenetic tree suggested two major propagation routes into Japan. In southern Japan, the local varieties of cowpea and ssp. *sesquipedalis* may have been introduced directly from China or via Taiwan, because Yonakuni jima, one of the islands in Japan where the earlier exploration of food legume species was carried out, is separated from Taiwan by only 160 km, as reported by Nakano et al.^77^. In northern Japan, cowpeas migrated from Southeast Asia. However, documentation of cowpea’s arrival in Southeast Asia is limited. The close clustering of ssp. *sesquipedalis* accessions from Southeast Asia with those from Japan indicate a shared lineage. Overall, cowpea migration into Asia and Japan likely followed two routes: Africa-India-South Asia-China-East Asia-Japan, and Africa-India-South Asia-China-Southeast Asia-Japan, with subsequent independent movements from East and Southeast Asia to Japan. These findings highlight the significant role of these two cultivation regions in cowpea transmission to Japan.

## Conclusion

This study, involving the sequencing of 410 Asian cowpea accessions on a large scale, generated significant genomic data, unveiling the gene pool structure and evolutionary trajectory of cowpea migration into Asia. Our findings highlight extensive genetic mixing of West and East African accessions within the Asian population but limited genetic mixing of Asian accessions within the African population, reinforcing the evidence that cowpea was introduced from Africa into Asia. Notably, we identified a significant genetic diversity within germplasm collections sourced from Nepal and Japan compared to other regions, suggesting that accessions from these regions may serve as valuable reservoirs of underutilized alleles. These findings emphasize the importance of incorporating diverse genetic resources into breeding programs to strengthen cowpea improvement. Additionally, this research holds promise for identifying advantageous alleles in Asian cowpea accessions through genome-wide association studies, further advancing cowpea breeding efforts.

## Supplementary Information

Supplementary Information.

## Data Availability

All data generated in this study, which underpins the conclusions drawn, have been included within this published article.
