# Supplementary material for: Genetic diversity and structure of Asian cowpea germplasm
Source: Sci Rep. 2025 Jul 31;15:27909. doi: 10.1038/s41598-025-13511-4 (PMC12313949; doi:10.1038/s41598-025-13511-4)
Supplement: Supplementary file 1 — Supplementary Information. [file 41598_2025_13511_MOESM1_ESM.pdf]

Supplementary Table 1.

Characterization of 410 cowpea germplasm accessions obtained from NARO Genebank

Lines	Accession ID	Subspecies	Status	Origin	Region
TOT1	53837	*unguiculata*	Cultivar	Japan	East Asia
TOT2	53838	*unguiculata*	Cultivar	Japan	East Asia
TOT3	53841	*unguiculata*	Cultivar	Japan	East Asia
TOT4	53844	*unguiculata*	Cultivar	Japan	East Asia
TOT5	53845	*unguiculata*	Cultivar	Japan	East Asia
TOT6	53846	*unguiculata*	Cultivar	Japan	East Asia
TOT7	53847	*unguiculata*	Cultivar	Japan	East Asia
TOT8	53849	*unguiculata*	Cultivar	Japan	East Asia
TOT9	87822	*unguiculata*	Cultivar	Japan	East Asia
TOT10	74825	*unguiculata*	Cultivar	Japan	East Asia
TOT11	74826	*unguiculata*	Cultivar	Japan	East Asia
TOT12	74827	*unguiculata*	Cultivar	Japan	East Asia
TOT13	74828	*unguiculata*	Cultivar	Japan	East Asia
TOT14	74829	*unguiculata*	Cultivar	Japan	East Asia
TOT15	78861	*unguiculata*	Cultivar	Japan	East Asia
TOT16	80032	*unguiculata*	Cultivar	Japan	East Asia
TOT17	80033	*unguiculata*	Cultivar	Japan	East Asia
TOT18	80034	*unguiculata*	Cultivar	Japan	East Asia
TOT19	80035	*unguiculata*	Cultivar	Japan	East Asia
TOT20	89096	*unguiculata*	Cultivar	Japan	East Asia
TOT21	89097	*unguiculata*	Cultivar	Japan	East Asia
TOT22	201494	*unguiculata*	Cultivar	Japan	East Asia
TOT23	201497	*unguiculata*	Cultivar	Japan	East Asia
TOT24	201524	*unguiculata*	Cultivar	Japan	East Asia
TOT25	201531	*unguiculata*	Cultivar	Japan	East Asia
TOT26	212390	*unguiculata*	Cultivar	Japan	East Asia
TOT27	212391	*unguiculata*	Cultivar	Japan	East Asia
TOT28	212392	*unguiculata*	Cultivar	Japan	East Asia
TOT29	259763	*unguiculata*	Cultivar	Japan	East Asia
TOT30	259764	*unguiculata*	Cultivar	Japan	East Asia
TOT31	76525	*unguiculata*	Cultivar	Japan	East Asia
TOT32	76621	*unguiculata*	Cultivar	Japan	East Asia
TOT33	31615	*sesquipedalis*	Cultivar	Japan	East Asia
TOT34	110723	*unguiculata*	Cultivar	Japan	East Asia
TOT35	110719	*unguiculata*	Cultivar	Japan	East Asia
TOT36	238612	*unguiculata*	Cultivar	Japan	East Asia
TOT37	252189	*unguiculata*	Cultivar	Japan	East Asia
TOT38	252226	*unguiculata*	Cultivar	Japan	East Asia
TOT39	31624	*unguiculata*	Cultivar	Japan	East Asia
TOT40	31621	*unguiculata*	Cultivar	Japan	East Asia
TOT41	110726	*unguiculata*	Cultivar	Japan	East Asia
TOT42	110727	*unguiculata*	Cultivar	Japan	East Asia

TOT43	240325	*unguiculata*	Cultivar	Japan	East Asia
TOT44	240326	*unguiculata*	Cultivar	Japan	East Asia
TOT45	87823	*unguiculata*	Cultivar	Japan	East Asia
TOT46	226773	*unguiculata*	Cultivar	Japan	East Asia
TOT47	226811	*unguiculata*	Cultivar	Japan	East Asia
TOT48	231294	*unguiculata*	Cultivar	Japan	East Asia
TOT49	233162	*unguiculata*	Cultivar	Japan	East Asia
TOT50	232269	*unguiculata*	Cultivar	Japan	East Asia
TOT51	232270	*unguiculata*	Cultivar	Japan	East Asia
TOT52	232271	*unguiculata*	Cultivar	Japan	East Asia
TOT53	78862	*unguiculata*	Cultivar	Japan	East Asia
TOT54	78863	*unguiculata*	Cultivar	Japan	East Asia
TOT55	78865	*unguiculata*	Cultivar	Japan	East Asia
TOT56	78866	*unguiculata*	Cultivar	Japan	East Asia
TOT57	78867	*unguiculata*	Cultivar	Japan	East Asia
TOT58	78868	*unguiculata*	Cultivar	Japan	East Asia
TOT59	78869	*unguiculata*	Cultivar	Japan	East Asia
TOT60	78870	*unguiculata*	Cultivar	Japan	East Asia
TOT61	78871	*unguiculata*	Cultivar	Japan	East Asia
TOT62	78872	*unguiculata*	Cultivar	Japan	East Asia
TOT63	78873	*unguiculata*	Cultivar	Japan	East Asia
TOT64	78874	*unguiculata*	Cultivar	Japan	East Asia
TOT65	78875	*unguiculata*	Cultivar	Japan	East Asia
TOT66	78876	*unguiculata*	Cultivar	Japan	East Asia
TOT67	78877	*unguiculata*	Cultivar	Japan	East Asia
TOT68	78878	*unguiculata*	Cultivar	Japan	East Asia
TOT69	78879	*unguiculata*	Cultivar	Japan	East Asia
TOT70	78880	*unguiculata*	Cultivar	Japan	East Asia
TOT71	78881	*unguiculata*	Cultivar	Japan	East Asia
TOT72	78882	*unguiculata*	Cultivar	Japan	East Asia
TOT73	78883	*unguiculata*	Cultivar	Japan	East Asia
TOT74	78884	*unguiculata*	Cultivar	Japan	East Asia
TOT75	78885	*unguiculata*	Cultivar	Japan	East Asia
TOT76	78886	*unguiculata*	Cultivar	Japan	East Asia
TOT77	78887	*unguiculata*	Cultivar	Japan	East Asia
TOT78	78888	*unguiculata*	Cultivar	Japan	East Asia
TOT79	78889	*unguiculata*	Cultivar	Japan	East Asia
TOT80	78890	*unguiculata*	Cultivar	Japan	East Asia
TOT81	78891	*unguiculata*	Cultivar	Japan	East Asia
TOT82	78892	*unguiculata*	Cultivar	Japan	East Asia
TOT83	85514	*unguiculata*	Cultivar	Japan	East Asia
TOT84	85515	*unguiculata*	Cultivar	Japan	East Asia
TOT85	85516	*unguiculata*	Cultivar	Japan	East Asia
TOT86	85517	*unguiculata*	Cultivar	Japan	East Asia
TOT87	85518	*unguiculata*	Cultivar	Japan	East Asia
TOT88	85519	*unguiculata*	Cultivar	Japan	East Asia

TOT89	85520	*unguiculata*	Cultivar	Japan	East Asia
TOT90	85521	*unguiculata*	Cultivar	Japan	East Asia
TOT91	85522	*unguiculata*	Cultivar	Japan	East Asia
TOT92	110721	*unguiculata*	Cultivar	Japan	East Asia
TOT93	110722	*unguiculata*	Cultivar	Japan	East Asia
TOT94	200976	*unguiculata*	Cultivar	Japan	East Asia
TOT95	53840	*unguiculata*	Cultivar	Japan	East Asia
TOT96	76622	*unguiculata*	Cultivar	Japan	East Asia
TOT97	31616	*sesquipedalis*	Cultivar	Japan	East Asia
TOT98	201431	*unguiculata*	Cultivar	Japan	East Asia
TOT99	81544	*unguiculata*	Cultivar	Japan	East Asia
TOT100	81571	*unguiculata*	Cultivar	Japan	East Asia
TOT101	81572	*unguiculata*	Cultivar	Japan	East Asia
TOT102	81573	*unguiculata*	Cultivar	Japan	East Asia
TOT103	81575	*unguiculata*	Cultivar	Japan	East Asia
TOT104	81577	*unguiculata*	Cultivar	Japan	East Asia
TOT105	81578	*unguiculata*	Cultivar	Japan	East Asia
TOT106	237059	*unguiculata*	Cultivar	Japan	East Asia
TOT107	237062	*unguiculata*	Cultivar	Japan	East Asia
TOT108	247310	*unguiculata*	Cultivar	Japan	East Asia
TOT109	247323	*unguiculata*	Cultivar	Japan	East Asia
TOT110	247339	*unguiculata*	Cultivar	Japan	East Asia
TOT111	247353	*unguiculata*	Cultivar	Japan	East Asia
TOT112	246136	*unguiculata*	Cultivar	Japan	East Asia
TOT113	246137	*unguiculata*	Cultivar	Japan	East Asia
TOT114	246478	*unguiculata*	Cultivar	Japan	East Asia
TOT115	31622	*unguiculata*	Cultivar	Japan	East Asia
TOT116	110724	*unguiculata*	Cultivar	Japan	East Asia
TOT117	110725	*unguiculata*	Cultivar	Japan	East Asia
TOT118	73010	*unguiculata*	Cultivar	Japan	East Asia
TOT119	110348	*unguiculata*	Cultivar	Japan	East Asia
TOT120	110353	*unguiculata*	Cultivar	Japan	East Asia
TOT121	220129	*unguiculata*	Cultivar	Laos	Southeast Asia
TOT122	222398	*unguiculata*	Cultivar	Laos	Southeast Asia
TOT123	224423	*unguiculata*	Cultivar	Laos	Southeast Asia
TOT124	224448	*unguiculata*	Cultivar	Laos	Southeast Asia
TOT125	226643	*unguiculata*	Cultivar	Laos	Southeast Asia
TOT126	226694	*unguiculata*	Cultivar	Laos	Southeast Asia
TOT127	226697	*unguiculata*	Cultivar	Laos	Southeast Asia
TOT128	226699	*unguiculata*	Cultivar	Laos	Southeast Asia
TOT129	230754	*unguiculata*	Cultivar	Laos	Southeast Asia
TOT130	230757	*unguiculata*	Cultivar	Laos	Southeast Asia
TOT131	230759	*unguiculata*	Cultivar	Laos	Southeast Asia
TOT132	230760	*unguiculata*	Cultivar	Laos	Southeast Asia
TOT134	230764	*unguiculata*	Cultivar	Laos	Southeast Asia
TOT135	233355	*unguiculata*	Cultivar	Laos	Southeast Asia

TOT136	233356	*unguiculata*	Cultivar	Laos	Southeast Asia
TOT137	42922	*unguiculata*	Cultivar	Nepal	South Asia
TOT138	42923	*unguiculata*	Cultivar	Nepal	South Asia
TOT139	42924	*unguiculata*	Cultivar	Nepal	South Asia
TOT140	42926	*unguiculata*	Cultivar	Nepal	South Asia
TOT141	42927	*cylindrica*	Cultivar	Nepal	South Asia
TOT142	42928	*unguiculata*	Cultivar	Nepal	South Asia
TOT143	42929	*unguiculata*	Cultivar	Nepal	South Asia
TOT144	42930	*unguiculata*	Cultivar	Nepal	South Asia
TOT145	42931	*unguiculata*	Cultivar	Nepal	South Asia
TOT146	42932	*unguiculata*	Cultivar	Nepal	South Asia
TOT147	74830	*unguiculata*	Cultivar	Nepal	South Asia
TOT148	85401	*unguiculata*	Cultivar	Nepal	South Asia
TOT149	85402	*unguiculata*	Cultivar	Nepal	South Asia
TOT150	85403	*unguiculata*	Cultivar	Nepal	South Asia
TOT151	85404	*unguiculata*	Cultivar	Nepal	South Asia
TOT152	85405	*unguiculata*	Cultivar	Nepal	South Asia
TOT153	85406	*unguiculata*	Cultivar	Nepal	South Asia
TOT154	85407	*unguiculata*	Cultivar	Nepal	South Asia
TOT155	85408	*unguiculata*	Cultivar	Nepal	South Asia
TOT156	85409	*unguiculata*	Cultivar	Nepal	South Asia
TOT157	85410	*unguiculata*	Cultivar	Nepal	South Asia
TOT158	85411	*unguiculata*	Cultivar	Nepal	South Asia
TOT159	85412	*unguiculata*	Cultivar	Nepal	South Asia
TOT160	85413	*unguiculata*	Cultivar	Nepal	South Asia
TOT161	85414	*unguiculata*	Cultivar	Nepal	South Asia
TOT162	85415	*unguiculata*	Cultivar	Nepal	South Asia
TOT163	85416	*unguiculata*	Cultivar	Nepal	South Asia
TOT164	85417	*unguiculata*	Cultivar	Nepal	South Asia
TOT165	85418	*unguiculata*	Cultivar	Nepal	South Asia
TOT166	85419	*unguiculata*	Cultivar	Nepal	South Asia
TOT168	85421	*unguiculata*	Cultivar	Nepal	South Asia
TOT169	85422	*unguiculata*	Cultivar	Nepal	South Asia
TOT170	85423	*unguiculata*	Cultivar	Nepal	South Asia
TOT171	85424	*unguiculata*	Cultivar	Nepal	South Asia
TOT172	85425	*unguiculata*	Cultivar	Nepal	South Asia
TOT173	85426	*sesquipedalis*	Cultivar	Nepal	South Asia
TOT174	85427	*unguiculata*	Cultivar	Nepal	South Asia
TOT175	85428	*sesquipedalis*	Cultivar	Nepal	South Asia
TOT176	85429	*unguiculata*	Cultivar	Nepal	South Asia
TOT177	85430	*unguiculata*	Cultivar	Nepal	South Asia
TOT178	85431	*unguiculata*	Cultivar	Nepal	South Asia
TOT179	85432	*unguiculata*	Cultivar	Nepal	South Asia
TOT180	85433	*unguiculata*	Cultivar	Nepal	South Asia
TOT181	85434	*unguiculata*	Cultivar	Nepal	South Asia
TOT182	85435	*unguiculata*	Cultivar	Nepal	South Asia

TOT183	85436	*unguiculata*	Cultivar	Nepal	South Asia
TOT184	85437	*unguiculata*	Cultivar	Nepal	South Asia
TOT185	85438	*unguiculata*	Cultivar	Nepal	South Asia
TOT186	85439	*sesquipedalis*	Cultivar	Nepal	South Asia
TOT187	85440	*unguiculata*	Cultivar	Nepal	South Asia
TOT188	85441	*unguiculata*	Cultivar	Nepal	South Asia
TOT189	85442	*unguiculata*	Cultivar	Nepal	South Asia
TOT190	85443	*unguiculata*	Cultivar	Nepal	South Asia
TOT191	85444	*unguiculata*	Cultivar	Nepal	South Asia
TOT192	85445	*sesquipedalis*	Cultivar	Nepal	South Asia
TOT193	85446	*sesquipedalis*	Cultivar	Nepal	South Asia
TOT194	85447	*unguiculata*	Cultivar	Nepal	South Asia
TOT195	85448	*unguiculata*	Cultivar	Nepal	South Asia
TOT196	85449	*unguiculata*	Cultivar	Nepal	South Asia
TOT197	85450	*unguiculata*	Cultivar	Nepal	South Asia
TOT198	85451	*unguiculata*	Cultivar	Nepal	South Asia
TOT199	85452	*sesquipedalis*	Cultivar	Nepal	South Asia
TOT200	85453	*unguiculata*	Cultivar	Nepal	South Asia
TOT201	85454	*unguiculata*	Cultivar	Nepal	South Asia
TOT202	85455	*sesquipedalis*	Cultivar	Nepal	South Asia
TOT203	85456	*unguiculata*	Cultivar	Nepal	South Asia
TOT204	85457	*unguiculata*	Cultivar	Nepal	South Asia
TOT205	85458	*unguiculata*	Cultivar	Nepal	South Asia
TOT206	85459	*unguiculata*	Cultivar	Nepal	South Asia
TOT207	85460	*unguiculata*	Cultivar	Nepal	South Asia
TOT208	85461	*unguiculata*	Cultivar	Nepal	South Asia
TOT209	85462	*unguiculata*	Cultivar	Nepal	South Asia
TOT210	85463	*unguiculata*	Cultivar	Nepal	South Asia
TOT211	85464	*unguiculata*	Cultivar	Nepal	South Asia
TOT212	85465	*unguiculata*	Cultivar	Nepal	South Asia
TOT213	85466	*unguiculata*	Cultivar	Nepal	South Asia
TOT214	85467	*unguiculata*	Cultivar	Nepal	South Asia
TOT215	85468	*unguiculata*	Cultivar	Nepal	South Asia
TOT216	85469	*unguiculata*	Cultivar	Nepal	South Asia
TOT217	85470	*unguiculata*	Cultivar	Nepal	South Asia
TOT218	85471	*unguiculata*	Cultivar	Nepal	South Asia
TOT219	85472	*unguiculata*	Cultivar	Nepal	South Asia
TOT220	85473	*unguiculata*	Cultivar	Nepal	South Asia
TOT221	85474	*unguiculata*	Cultivar	Nepal	South Asia
TOT222	85475	*unguiculata*	Cultivar	Nepal	South Asia
TOT223	85476	*unguiculata*	Cultivar	Nepal	South Asia
TOT224	85477	*unguiculata*	Cultivar	Nepal	South Asia
TOT225	85478	*unguiculata*	Cultivar	Nepal	South Asia
TOT226	97584	*unguiculata*	Cultivar	Nepal	South Asia
TOT227	100325	*unguiculata*	Cultivar	Nepal	South Asia
TOT228	233557	*unguiculata*	Cultivar	Nepal	South Asia

TOT229	74831	*unguiculata*	Cultivar	Pakistan	South Asia
TOT230	74832	*unguiculata*	Cultivar	Pakistan	South Asia
TOT231	74833	*cylindrica*	Cultivar	Pakistan	South Asia
TOT232	74834	*cylindrica*	Cultivar	Pakistan	South Asia
TOT233	74836	*unguiculata*	Cultivar	Pakistan	South Asia
TOT234	74837	*unguiculata*	Cultivar	Pakistan	South Asia
TOT235	74838	*unguiculata*	Cultivar	Pakistan	South Asia
TOT236	74839	*unguiculata*	Cultivar	Pakistan	South Asia
TOT237	74840	*unguiculata*	Cultivar	Pakistan	South Asia
TOT238	74841	*unguiculata*	Cultivar	Pakistan	South Asia
TOT239	74842	*unguiculata*	Cultivar	Pakistan	South Asia
TOT240	74843	*unguiculata*	Cultivar	Pakistan	South Asia
TOT241	74844	*unguiculata*	Cultivar	Pakistan	South Asia
TOT242	74846	*unguiculata*	Cultivar	Pakistan	South Asia
TOT243	74847	*unguiculata*	Cultivar	Pakistan	South Asia
TOT244	74848	*unguiculata*	Cultivar	Pakistan	South Asia
TOT245	74849	*unguiculata*	Cultivar	Pakistan	South Asia
TOT246	74850	*unguiculata*	Cultivar	Pakistan	South Asia
TOT247	103120	*unguiculata*	Cultivar	Pakistan	South Asia
TOT248	103123	*unguiculata*	Cultivar	Pakistan	South Asia
TOT249	103133	*unguiculata*	Cultivar	Pakistan	South Asia
TOT250	104292	*unguiculata*	Cultivar	Pakistan	South Asia
TOT251	104293	*unguiculata*	Cultivar	Pakistan	South Asia
TOT252	104294	*unguiculata*	Cultivar	Pakistan	South Asia
TOT253	104295	*unguiculata*	Cultivar	Pakistan	South Asia
TOT254	104297	*unguiculata*	Cultivar	Pakistan	South Asia
TOT255	104298	*unguiculata*	Cultivar	Pakistan	South Asia
TOT256	104300	*unguiculata*	Cultivar	Pakistan	South Asia
TOT257	104302	*unguiculata*	Cultivar	Pakistan	South Asia
TOT258	104304	*unguiculata*	Cultivar	Pakistan	South Asia
TOT259	104305	*unguiculata*	Cultivar	Pakistan	South Asia
TOT260	104306	*unguiculata*	Cultivar	Pakistan	South Asia
TOT261	104307	*unguiculata*	Cultivar	Pakistan	South Asia
TOT262	105402	*unguiculata*	Cultivar	Pakistan	South Asia
TOT263	110792	*unguiculata*	Cultivar	Pakistan	South Asia
TOT264	233325	*unguiculata*	Cultivar	Pakistan	South Asia
TOT265	233327	*unguiculata*	Cultivar	Pakistan	South Asia
TOT266	233331	*unguiculata*	Cultivar	Pakistan	South Asia
TOT267	236742	*unguiculata*	Cultivar	Pakistan	South Asia
TOT268	236744	*unguiculata*	Cultivar	Pakistan	South Asia
TOT269	237629	*unguiculata*	Cultivar	Pakistan	South Asia
TOT270	237630	*unguiculata*	Cultivar	Pakistan	South Asia
TOT271	237631	*unguiculata*	Cultivar	Pakistan	South Asia
TOT273	247430	*unguiculata*	Cultivar	Pakistan	South Asia
TOT274	247431	*unguiculata*	Cultivar	Pakistan	South Asia

TOT275	222494	*sesquipedalis*	Cultivar	Papua New Guinea	Oceania
TOT276	81550	*unguiculata*	Cultivar	Sri Lanka	South Asia
TOT277	81551	*unguiculata*	Cultivar	Sri Lanka	South Asia
TOT278	81552	*unguiculata*	Cultivar	Sri Lanka	South Asia
TOT279	81553	*unguiculata*	Cultivar	Sri Lanka	South Asia
TOT280	81554	*unguiculata*	Cultivar	Sri Lanka	South Asia
TOT281	81592	*unguiculata*	Cultivar	Sri Lanka	South Asia
TOT282	81593	*unguiculata*	Cultivar	Sri Lanka	South Asia
TOT283	81594	*unguiculata*	Cultivar	Sri Lanka	South Asia
TOT284	81595	*unguiculata*	Cultivar	Sri Lanka	South Asia
TOT285	81596	*unguiculata*	Cultivar	Sri Lanka	South Asia
TOT286	81597	*unguiculata*	Cultivar	Sri Lanka	South Asia
TOT287	81600	*unguiculata*	Cultivar	Sri Lanka	South Asia
TOT288	81604	*unguiculata*	Cultivar	Sri Lanka	South Asia
TOT289	41278	*unguiculata*	Cultivar	Thailand	Southeast Asia
TOT290	41279	*unguiculata*	Cultivar	Thailand	Southeast Asia
TOT291	41280	*unguiculata*	Cultivar	Thailand	Southeast Asia
TOT292	41281	*unguiculata*	Cultivar	Thailand	Southeast Asia
TOT293	41282	*unguiculata*	Cultivar	Thailand	Southeast Asia
TOT294	41283	*sesquipedalis*	Cultivar	Thailand	Southeast Asia
TOT295	81549	*unguiculata*	Cultivar	Thailand	Southeast Asia
TOT296	86824	*unguiculata*	Cultivar	Ethiopia	East Africa
TOT297	86825	*unguiculata*	Cultivar	Ethiopia	East Africa
TOT298	86826	*unguiculata*	Cultivar	Ethiopia	East Africa
TOT299	97459	*unguiculata*	Cultivar	Ethiopia	East Africa
TOT300	31654	*unguiculata*	Cultivar	Nigeria	West Africa
TOT301	31655	*unguiculata*	Cultivar	Nigeria	West Africa
TOT302	31656	*unguiculata*	Cultivar	Nigeria	West Africa
TOT303	31657	*unguiculata*	Cultivar	Nigeria	West Africa
TOT304	31658	*unguiculata*	Cultivar	Nigeria	West Africa
TOT305	31659	*unguiculata*	Cultivar	Nigeria	West Africa
TOT306	31660	*unguiculata*	Cultivar	Nigeria	West Africa
TOT307	31661	*unguiculata*	Cultivar	Nigeria	West Africa
TOT308	31662	*unguiculata*	Cultivar	Nigeria	West Africa
TOT309	31663	*unguiculata*	Cultivar	Nigeria	West Africa
TOT310	31664	*unguiculata*	Cultivar	Nigeria	West Africa
TOT311	31665	*unguiculata*	Cultivar	Nigeria	West Africa
TOT312	31666	*unguiculata*	Cultivar	Nigeria	West Africa
TOT313	31667	*unguiculata*	Cultivar	Nigeria	West Africa
TOT314	31668	*unguiculata*	Cultivar	Nigeria	West Africa
TOT315	31670	*unguiculata*	Cultivar	Nigeria	West Africa
TOT316	31671	*unguiculata*	Cultivar	Nigeria	West Africa
TOT317	31672	*unguiculata*	Cultivar	Nigeria	West Africa
TOT318	31673	*unguiculata*	Cultivar	Nigeria	West Africa
TOT319	31674	*unguiculata*	Cultivar	Nigeria	West Africa

TOT320	31677	*unguiculata*	Cultivar	Nigeria	West Africa
TOT321	86875	*unguiculata*	Cultivar	Sudan	Northeast Africa
TOT322	86876	*unguiculata*	Cultivar	Sudan	Northeast Africa
TOT323	86877	*unguiculata*	Cultivar	Sudan	Northeast Africa
TOT324	86878	*unguiculata*	Cultivar	Sudan	Northeast Africa
TOT325	86879	*unguiculata*	Cultivar	Sudan	Northeast Africa
TOT326	31625	*unguiculata*	Cultivar	China	East Asia
TOT327	80029	*unguiculata*	Cultivar	China	East Asia
TOT328	97441	*unguiculata*	Cultivar	China	East Asia
TOT329	132380	*unguiculata*	Cultivar	China	East Asia
TOT330	236697	*unguiculata*	Cultivar	China	East Asia
TOT331	236698	*unguiculata*	Cultivar	China	East Asia
TOT332	236702	*unguiculata*	Cultivar	China	East Asia
TOT333	236745	*unguiculata*	Cultivar	China	East Asia
TOT334	81555	*unguiculata*	Cultivar	Ghana	West Africa
TOT335	81556	*unguiculata*	Cultivar	Ghana	West Africa
TOT336	81557	*unguiculata*	Cultivar	Ghana	West Africa
TOT337	81558	*unguiculata*	Cultivar	Ghana	West Africa
TOT338	81559	*unguiculata*	Cultivar	Ghana	West Africa
TOT339	81560	*unguiculata*	Cultivar	Ghana	West Africa
TOT340	81561	*unguiculata*	Cultivar	Ghana	West Africa
TOT341	81562	*unguiculata*	Cultivar	Ghana	West Africa
TOT342	81563	*unguiculata*	Cultivar	Ghana	West Africa
TOT343	81564	*unguiculata*	Cultivar	Ghana	West Africa
TOT344	81565	*unguiculata*	Cultivar	Ghana	West Africa
TOT345	81566	*unguiculata*	Cultivar	Ghana	West Africa
TOT346	81567	*unguiculata*	Cultivar	Ghana	West Africa
TOT347	81568	*unguiculata*	Cultivar	Ghana	West Africa
TOT348	81569	*unguiculata*	Cultivar	Ghana	West Africa
TOT349	81570	*unguiculata*	Cultivar	Ghana	West Africa
TOT350	78860	*unguiculata*	Cultivar	Russia	Europe
TOT351	233358	*unguiculata*	Cultivar	Benin	West Africa
TOT352	233359	*unguiculata*	Cultivar	Benin	West Africa
TOT353	233360	*unguiculata*	Cultivar	Benin	West Africa
TOT354	233361	*unguiculata*	Cultivar	Benin	West Africa
TOT355	233366	*unguiculata*	Cultivar	Benin	West Africa
TOT356	233372	*unguiculata*	Cultivar	Benin	West Africa
TOT357	233373	*unguiculata*	Cultivar	Benin	West Africa
TOT358	233375	*unguiculata*	Cultivar	Benin	West Africa
TOT359	233387	*unguiculata*	Cultivar	Benin	West Africa
TOT360	233390	*unguiculata*	Cultivar	Benin	West Africa
TOT361	89230	*sesquipedalis*	Cultivar	Malaysia	Southeast Asia
TOT362	89231	*sesquipedalis*	Cultivar	Malaysia	Southeast Asia
TOT363	89232	*sesquipedalis*	Cultivar	Malaysia	Southeast Asia
TOT364	89285	*sesquipedalis*	Cultivar	Malaysia	Southeast Asia
TOT365	244395	*unguiculata*	Cultivar	Cambodia	Southeast Asia

TOT366	244396	*unguiculata*	Cultivar	Cambodia	Southeast Asia
TOT367	244404	*unguiculata*	Cultivar	Cambodia	Southeast Asia
TOT368	247213	*unguiculata*	Cultivar	Cambodia	Southeast Asia
TOT370	247239	*unguiculata*	Cultivar	Cambodia	Southeast Asia
TOT371	251271	*unguiculata*	Cultivar	Cambodia	Southeast Asia
TOT372	251276	*unguiculata*	Cultivar	Cambodia	Southeast Asia
TOT373	251282	*unguiculata*	Cultivar	Cambodia	Southeast Asia
TOT375	251304	*unguiculata*	Cultivar	Cambodia	Southeast Asia
TOT376	251306	*unguiculata*	Cultivar	Cambodia	Southeast Asia
TOT377	251310	*unguiculata*	Cultivar	Cambodia	Southeast Asia
TOT378	252340	*unguiculata*	Cultivar	Cambodia	Southeast Asia
TOT381	252354	*unguiculata*	Cultivar	Cambodia	Southeast Asia
TOT382	252361	*unguiculata*	Cultivar	Cambodia	Southeast Asia
TOT383	252362	*unguiculata*	Cultivar	Cambodia	Southeast Asia
TOT384	252374	*unguiculata*	Cultivar	Cambodia	Southeast Asia
TOT385	252379	*unguiculata*	Cultivar	Cambodia	Southeast Asia
TOT386	252381	*unguiculata*	Cultivar	Cambodia	Southeast Asia
TOT387	257438	*unguiculata*	Cultivar	Japan	East Asia
TOT388	254624	*unguiculata*	Cultivar	Japan	East Asia
TOT389	254625	*unguiculata*	Cultivar	Japan	East Asia
TOT390	254635	*unguiculata*	Cultivar	Japan	East Asia
TOT391	211966	*sesquipedalis*	Cultivar	Myanmar	Southeast Asia
TOT393	232352	*unguiculata*	Cultivar	Myanmar	Southeast Asia
TOT394	243043	*unguiculata*	Cultivar	Myanmar	Southeast Asia
TOT395	243048	*sesquipedalis*	Cultivar	Myanmar	Southeast Asia
TOT397	243063	*sesquipedalis*	Cultivar	Myanmar	Southeast Asia
TOT398	252261	*sesquipedalis*	Cultivar	Myanmar	Southeast Asia
TOT399	252307	*sesquipedalis*	Cultivar	Myanmar	Southeast Asia
TOT400	252321	*unguiculata*	Cultivar	Myanmar	Southeast Asia
TOT401	254840	*unguiculata*	Cultivar	Myanmar	Southeast Asia
TOT402	254846	*sesquipedalis*	Cultivar	Myanmar	Southeast Asia
TOT403	257502	*unguiculata*	Cultivar	Nepal	South Asia
TOT404	257503	*unguiculata*	Cultivar	Nepal	South Asia
TOT405	257504	*unguiculata*	Cultivar	Nepal	South Asia
TOT406	257539	*unguiculata*	Cultivar	Nepal	South Asia
TOT407	257545	*unguiculata*	Cultivar	Nepal	South Asia
TOT408	257552	*unguiculata*	Cultivar	Nepal	South Asia
TOT409	257553	*unguiculata*	Cultivar	Nepal	South Asia
TOT410	257554	*unguiculata*	Cultivar	Nepal	South Asia
TOT411	257556	*unguiculata*	Cultivar	Nepal	South Asia
TOT412	257562	*unguiculata*	Cultivar	Nepal	South Asia
TOT413	258027	*unguiculata*	Cultivar	Nepal	South Asia
TOT414	258028	*unguiculata*	Cultivar	Nepal	South Asia
TOT415	IT86D-1010	*unguiculata*	Cultivar	Nigeria	West Africa
TOT416	IT97K-499-35	*unguiculata*	Cultivar	Nigeria	West Africa
TOT417	Sasaque	*unguiculata*	Cultivar	Japan	East Asia

TOT418	Michiko	*unguiculata*	Cultivar	Japan	East Asia
TOTS139	NI1419	*unguiculata*	Wild	Botswana	South Africa

TOT1 – TOT414 were obtained from the NARO genebank: https://www.gene.affrc.go.jp/index_en.php

TOT415 and TOT416 were obtained from the International Institute of Tropical Agriculture (IITA), Nigeria: https://www.iita.org/

TOTS139 was obtained from Meise Botanic Garden: https://www.plantentuinmeise.be/en/

Supplementary Table 2.

Analysis of molecular variance (AMOVA) results among populations and within accessions

**Source of variation**	**df**	**SS**	**MS**	**Est. Var.**	**%**	***p*-Value**
Among Pops	14	5036.210	359.729	13.111	20%	0.001
Within Pops	388	20008.795	51.569	51.569	80%	0.001
Total	402	25045.005		64.680	100%	
Nm	1.97					

df: degree of freedom, SS: sum of squares, MS: mean square, Est. Var.: estimated variance, % = percentage variance, Nm: gene flow.

Supplementary Figure 1

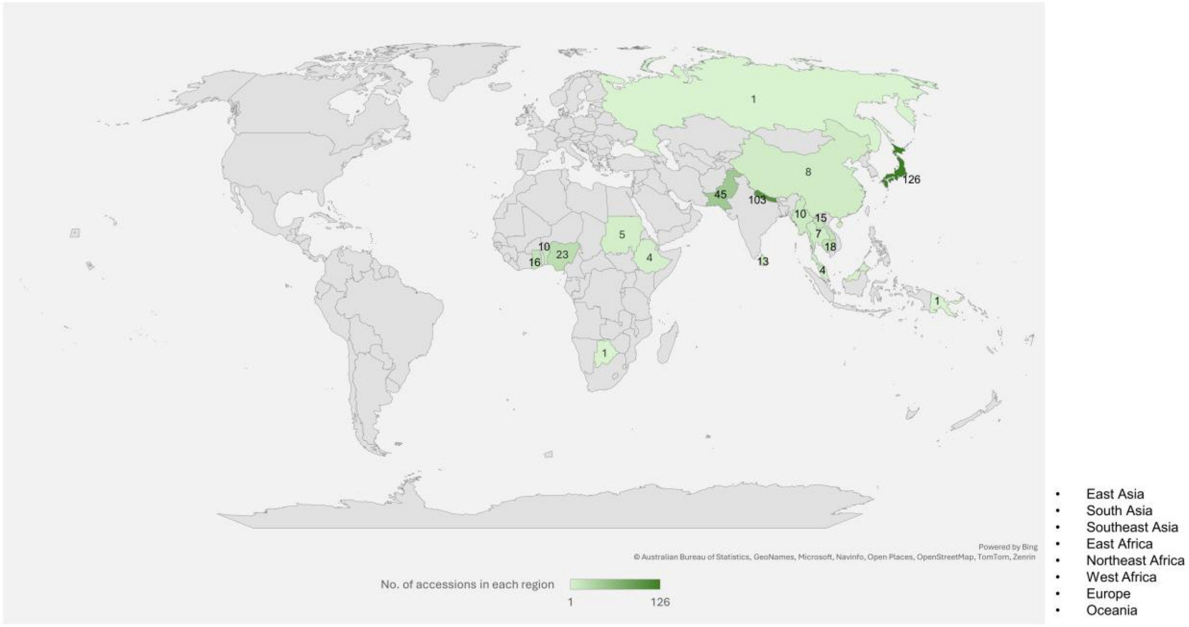

Supplementary Figure 1. Map showing the different regions of Asia, Africa, Europe, and Oceania from where cowpea germplasm was collected for assessing genetic diversity and divergence. The sample sizes are represented by a scale bar and numerical values as indicated on the regions. The map was generated using Excel.

Supplementary Figure 2

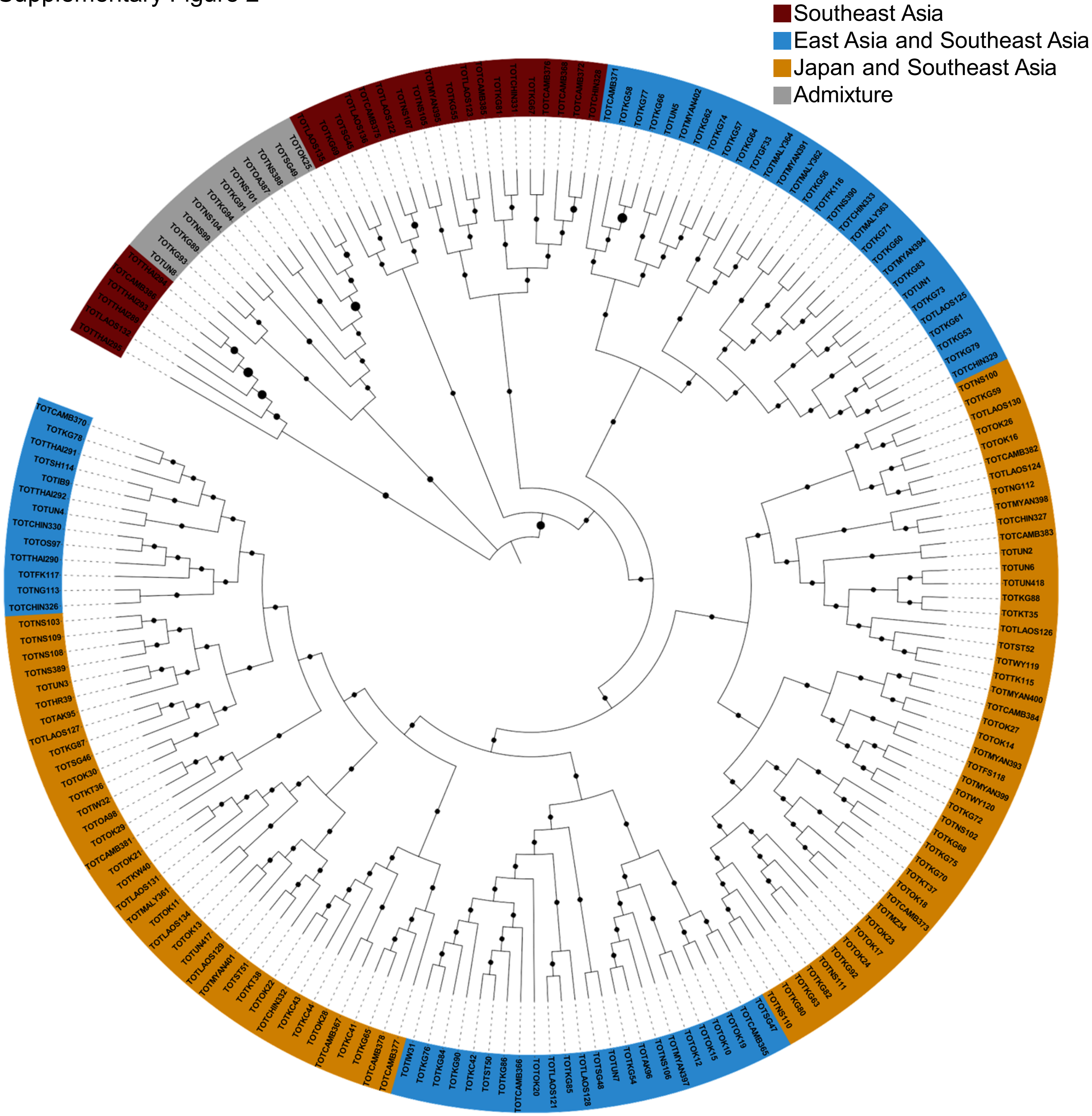

Supplementary Figure 2. Maximum-likelihood phylogenetic tree illustrating the evolutionary relationships among 188 cowpea accessions from East Asia and Southeast Asia, based on 6,612 markers. The clustering pattern reflects the geographical and cultivar-specific origin or domestication. Colored branches indicate germplasm sources: red for Southeast Asia, blue for East Asia (Japan and China), and Southeast Asia, yellow for Japan and Southeast Asia, and grey for admixed individuals. Black dots indicate boot strap values ranging from 0 to 1.
